# Cerebral involvement in a patient with Goodpasture's disease due to shortened induction therapy: a case report

**DOI:** 10.1186/1752-1947-3-120

**Published:** 2009-11-12

**Authors:** Christoph Preul, Jens Gerth, Sebastian Lang, Christoph Bergmeier, Otto W Witte, Gunter Wolf, Christoph Terborg

**Affiliations:** 1 Department of Neurology, University Hospital of Friedrich-Schiller University, 07740 Jena, Germany; 2 Department of Internal Medicine - III, University Hospital of Friedrich-Schiller University, 07740 Jena, Germany; 3 Department of Anesthesiology and Intensive Care Medicine, University Hospital of Friedrich-Schiller University, 07740 Jena, Germany

## Abstract

**Introduction:**

Goodpasture's disease is a rare immunological disease with formation of pathognomonic antibodies against renal and pulmonary basement membranes. Cerebral involvement has been reported in several cases in the literature, yet the pathogenetic mechanism is not entirely clear.

**Case presentation:**

A 21-year-old Caucasian man with Goodpasture's disease and end-stage renal disease presented with two generalized seizures after a period of mild cognitive disturbance. Blood pressure and routine laboratory tests did not exceed the patient's usual values, and examination of cerebrospinal fluid was unremarkable. Cerebral magnetic resonance imaging (MRI) revealed multiple cortical and subcortical lesions on fluid-attenuated inversion recovery sequences. Since antiglomerular basement membrane antibodies were found to be positive with high titers, plasmapheresis was started. In addition, cyclophosphamide pulse therapy was given on day 13. Encephalopathy and MRI lesions disappeared during this therapy, and antiglomerular basement membrane antibodies were significantly reduced. Previous immunosuppressive therapy was performed without corticosteroids and terminated early after 3 months.

The differential diagnostic considerations were cerebral vasculitis and posterior reversible encephalopathy syndrome. Vasculitis could be seen as an extrarenal manifestation of the underlying disease. Posterior reversible encephalopathy syndrome, on the other hand, can be triggered by immunosuppressive therapy and may appear without a hypertensive crisis.

**Conclusion:**

A combination of central nervous system symptoms with a positive antiglomerular basement membrane test in a patient with Goodpasture's disease should immediately be treated as an acute exacerbation of the disease with likely cross-reactivity of antibodies with the choroid plexus. In our patient, a discontinuous strategy of immunosuppressive therapy may have favored recurrence of Goodpasture's disease.

## Introduction

In Goodpasture's disease, a type II hypersensitivity reaction is present with antibody and T-lymphocyte reactivity to the NC1 domain of the alpha3 chain of type 4 collagen [1]. These specific antigens exist on the basement membranes of the kidney and pulmonary alveoli [2] but not on the basal membranes of the brain. However, the antigen has been found in the choroid plexus [3,4] and it has been shown that even normal individuals have low titers of antiglomerular basement membrane (anti-GBM) antibodies [5]. Although NC1 is expressed in the thymus, CD4+ cells can escape thymic deletion and participate in the disease. It is postulated that failure to develop tolerance to high-affinity peptides from this antigen is likely to be a consequence of the failure of antigen-presenting cells [1,6].

The usual treatments for Goodpasture's disease are administration of cyclophosphamide and prednisolone, and removal of pathogenic antibodies with plasmapheresis, as the activity of the disease correlates with the antibody level. The latter has drastically improved the prognosis and outcome in patients with Goodpasture's disease [7-9]. Maintaining therapy of oral prednisolone is recommended for at least 6 months, starting at a dose of 1 mg/kg daily, and continuously reducing it over the following 6 months.

## Case presentation

A 21-year-old Caucasian man with histologically proven (renal biopsy) Goodpasture's disease since spring 2006 was admitted to our hospital after two generalized tonic-clonic seizures with preceding neuropsychological symptoms of decreased alertness and slowed executive functions. The patient was found to be somnolent, with elevated blood pressure of 180/90 mmHg and a second generalized seizure. Aspiration during the seizure required intubation and mechanical ventilation until the third day after admission.

Regarding his past medical history, the patient was first treated for a rapid progressive glomerulonephritis (RPGN) in another hospital when Goodpasture's disease was diagnosed histologically through renal biopsy (linear deposition of immunoglobulins along the basement membrane) and detection of anti-GBM antibodies in the plasma. A cyclophosphamide pulse therapy was administered, but renal disease progressed and hemodialysis became necessary and the cyclophosphamide therapy was terminated. One month later, renal replacement therapy was switched to continuous ambulatory peritoneal dialysis. Four months later, the patient was readmitted because of a pulmonary complication with anemia due to tracheal suffusions and microbleeds in combination with a gastrointestinal reflux disease. Cyclophosphamide therapy was reintroduced with monthly administration of 1 g as a bolus, initially. Immunosuppressive treatment yielded good elimination of anti-GBM antibodies. However, a consequent immunosuppressive therapy of at least 6 months duration had never been maintained. In summary, the patient received three therapy cycles before admission to our hospital with the central nervous system symptoms, but neither cyclophosphamide nor steroids had been given on a regular basis.

**Figure 1:**
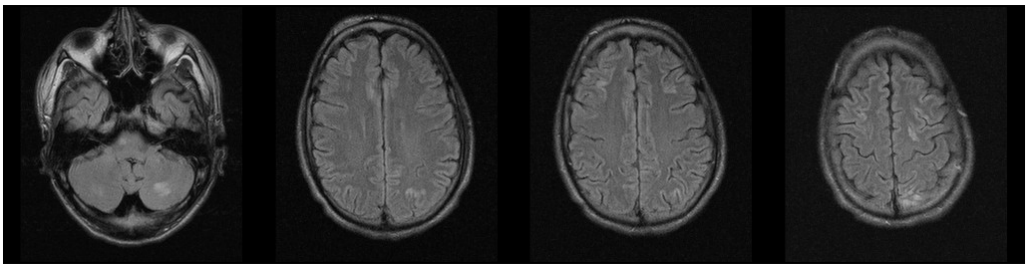
**Axial fluid-attenuated inversion recovery sequence upon the patient's admission revealed multiple edematous lesions in subcortical and juxtacortical white matter consistent with a vasculitis or posterior reversible encephalopathy syndrome**.

**Figure 2:**
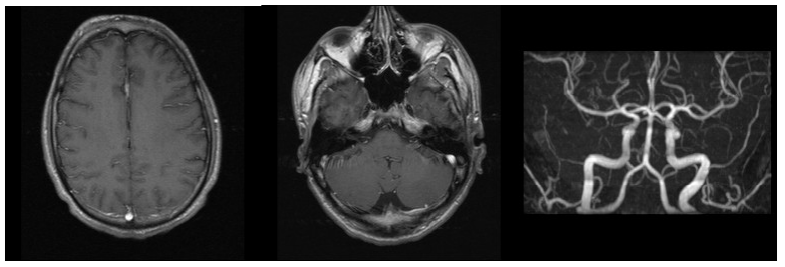
**(a, b) Contrast enhanced magnetic resonance imaging revealed non-enhancing lesions**. (c) Time-of-flight angiography did not detect cerebral vasculitis and showed normal vessel calibers.

Routine laboratory tests showed an elevation of creatinine (1107 μmol/l, normal value: 72-127 μmol/l) and serum urea (16.4 mmol/l, normal value: 3.0-9.2 mmol/l), while blood cell count, electrolytes, blood gas analysis and liver enzymes were normal. A chest X-ray was consistent with pneumonia after aspiration. Cranial computed tomography was normal. Cerebral magnetic resonance imaging (MRI) revealed multiple T2- and fluid-attenuated inversion recovery (FLAIR)-hyperintense lesions subcortically, predominantly located in the posterior dorsal and lateral white matter as well as in the cerebellar white matter, consistent with cerebral edema (Figures 1 and 2). The lesions did not enhance contrast media nor did they match a pattern of ischemic infarctions. The time-of-flight angiography did not detect vasculitic stenosis.

**Figure 3:**
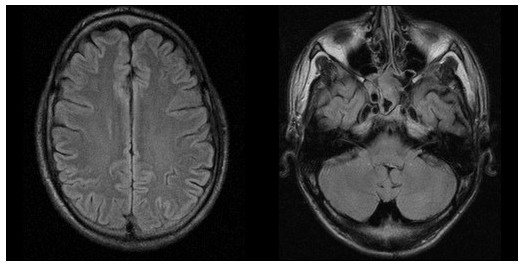
**During treatment, only residual signal alterations could be detected in an axial fluid-attenuated inversion recovery sequence**.

**Figure 4:**
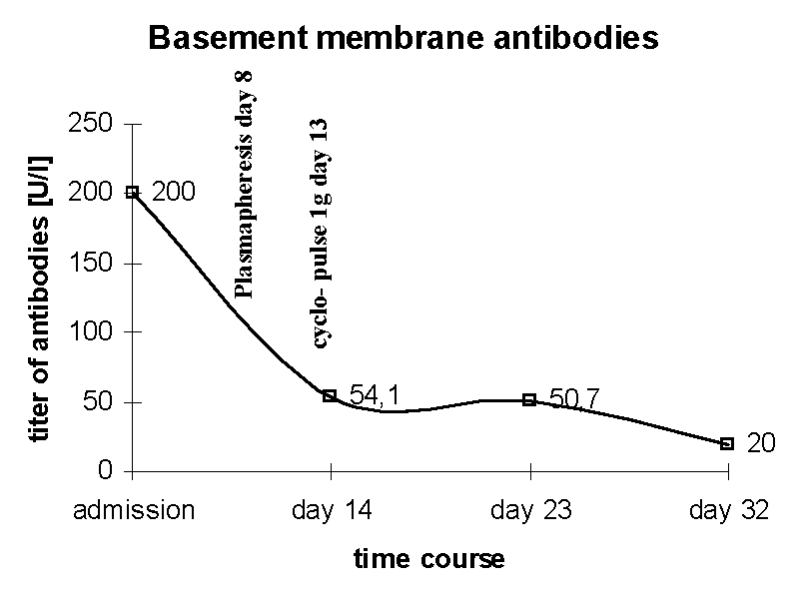
**Titer of antibodies against basement membranes measured at different time points during in-house treatment**. Delayed reduction of titer after cyclophosphamide matches with the half-life time of immunoglobulin G of 1-3 weeks. Plasmapheretic therapy was first applied at day 8 and then continued alternating with continuous veno-venous hemofiltration throughout the patient's treatment.

After extubation, the patient presented a mild psycho-syndrome with cognitive slowing and deficits in mnestic function. Apart from a positive Babinski sign on the left, his neurological state was normal. Due to the initial presentation with pneumonia, high-dose steroid therapy was not administered. Since anti-GBM antibodies were elevated (200 U/mL, normal value: <10 U/mL), plasmapheresis was started, alternating with continuous veno-venous hemofiltration. During this therapy, the psycho-syndrome resolved and the MR-morphological alterations almost diminished in parallel (Figure 3). Anti-GBM antibodies scaled down to 21 U/mL after five cycles of plasmapheresis initiated at day 6 (substitution of 4 litre albumin at each session) and one pulse of 1 g cyclophosphamide at day 13 (Figure 4). Antinuclear antibodies were negative at all times. The patient was dismissed in a neurologically and neuropsychologically healthy condition under continued oral cyclophosphamide therapy.

## Discussion

The three most likely differential diagnoses for this case were cerebral vasculitis, metabolic encephalopathy, and posterior reversible encephalopathy syndrome (PRES).

Cerebral vasculitis can occur with antineutrophil cytoplasmic antibodies (ANCA)-positive rheumatic diseases such as Wegener's granulomatosis or systemic lupus erythematosus [10]. In our patient, Goodpasture's disease was histologically established by kidney biopsy and proof of anti-GBM antibodies in circulation. ANCAs were never positive. There are case reports in the literature that report ANCA-negative cerebral vasculitic alterations coinciding with Goodpasture's disease [11,12], but the mechanism of cerebral involvement is not entirely clear.

A diagnosis of cerebral vasculitis was supported by the rapid response to treatment, although it was hampered by a negative MRI scan and a missing biopsy. Normal vessels on MRI, though, do not exclude small vessel vasculitis [13]. A conventional angiography was not performed in view of the potential side effects and the limited therapeutic consequences.

Differential diagnosis of metabolic encephalopathy seemed unlikely: liver enzymes were not significantly elevated, renal parameters were within the patient's usual range, and mistakes in performing peritoneal dialysis were anamnestically ruled out.

The most likely differential diagnosis for this patient is PRES. This is characterized by neurological alterations such as headache, seizures, focal neurological signs, and cerebral edema of the white matter predominantly in the posterior parts of the brain. Hypertension, immunosuppressive therapy, and uremia may be contributing factors. The MR-morphological signs of cerebral edema may be attributed to a loss of cerebral autoregulation [14]. PRES is more likely in patients with sepsis, and infections with Gram-positive organisms turned out to be a contributing factor [15]. Although clinical presentation, coincident immunosuppression, and the MR-morphological features match the diagnosis of PRES, no actual cause of the disease could be found, so this diagnosis was ruled out. Blood pressure was within the patient's normal range, and the symptoms and MR-morphological alterations resolved during plasmapheresis. A bacterium causing the pneumonia could not be isolated.

## Conclusion

In summary, our case is unique in that we took a differential diagnosis of cerebral vasculitis on the basis of a Goodpasture's disease into account. In an extensive meta-analysis for ANCA-associated vasculitides, it has been shown that pulsed immunosuppressive therapy with cyclophosphamide has a lower toxicity than continuous administration of this drug. Pulsed cyclophosphamide treatment seems to come at the cost of a higher rate of recurrence [9]. A discontinuous strategy of immunosuppressive therapy may have favored recurrence of Goodpasture's disease, and that the cerebral involvement can thus be seen as the third relapse of the disease.

## Abbreviations

ANCA: anti-neutrophil cytoplasmic antibodies; anti-GBM: anti-glomerular basement membrane; FLAIR: fluid-attenuated inversion recovery; MRI: magnetic resonance imaging; PRES: posterior reversible encephalopathy syndrome; RPGN: rapid progressive glomerulonephritis

## Consent

Written informed consent was obtained from the patient for publication of this case report and any accompanying images. A copy of the written consent is available for review by the Editor-in-Chief of this journal.

## Competing interests

The authors declare that they have no competing interests.

## Authors' contributions

CP and JG interpreted the patient data and clinical course regarding the neurological and renal disease. SL and CB contributed their extensive knowledge in intensive care medicine and plasmapheresis in particular. OWW, GW and CT were major contributors in discussing and writing the manuscript. All authors read and approved the final manuscript.
